# Understanding stakeholders’ experience with sickle cell disease by social media listening across Europe

**DOI:** 10.3389/fgene.2025.1629510

**Published:** 2025-09-29

**Authors:** Daniel Brás, Celeste Bento, Sathyaraj Asaithambi, Jyoti Chauhan, Ines Moital

**Affiliations:** ^1^ Novartis, Produtos Farmacêuticos SA, Porto Salvo, Portugal; ^2^ Comprehensive Health Research Centre (CHRC), Escola Nacional de Saúde Pública, Universidade NOVA de Lisboa, Lisboa, Portugal; ^3^ Department of Hematology, Centro Hospitalar e Universitário de Coimbra, Coimbra, Portugal; ^4^ Research Centre for Anthropology and Health (CIAS), Universidade Coimbra, Coimbra, Portugal; ^5^ Novartis Healthcare Pvt. Ltd., Hyderabad, India

**Keywords:** social media listening, sickle cell disease, caregiver, patient, quality of life

## Abstract

**Background:**

The use of social media platforms for sharing health-related information is on the rise. Sickle cell disease (SCD) affects millions of people worldwide. However, discussions by SCD stakeholders on social media remain unexplored. This study aimed to analyze discussions among SCD stakeholders on social media to understand their awareness of SCD and to explore their perceptions of the patient journey, hospitalizations and complications due to SCD, the impact of the disease on quality of life (QoL), and current unmet needs by using social media listening (SML).

**Methods:**

Data was retrospectively collected from April 2019 to April 2021 on SCD specific terms in 14 European countries from blogs, forums, and social networking sites (Twitter, public Facebook, YouTube, and Instagram). Advanced social media analytics tools, Talkwalker and Social Studio, were used for data aggregation and analysis. Conversations were filtered and contextualized through a 3-tier technique involving automated relevancy algorithms and manual review.

**Results:**

Of 317.9K conversations on SCD (93% Twitter), 945 posts on relevant patient-centric conversation were analyzed. Most patients were females (73%) and ≤30 years old (75%). Patient journey stages were addressed in 52% of conversations. Patient journey conversations were mainly regarding symptoms (56%) (mainly pain episodes, pain in general, and vaso-occlusive crises) and treatment (44%). Conversations on hospital visits or hospitalization mostly revolved around crises faced due to symptoms. Impact on QoL, especially emotional impact (56%), was also extensively discussed. Unmet needs were derived from 24% of the conversations, lack of awareness of SCD (42%) and lack of empathy and support from HCPs (24%) being the most frequent topics. Patients reported having their symptoms questioned or dismissed by healthcare professionals, which they attributed to racial bias.

**Conclusion:**

SML proves to be a useful tool for exploring the real experiences, concerns, and needs of SCD patients and other stakeholders. Analysis of SCD-related social media posts reveals that discussions mainly focus on symptoms, particularly pain, treatment, and the emotional impact of SCD on QoL. These insights are crucial for enhancing the management of SCD patients.

## Introduction

Sickle cell disease (SCD) is a genetic hematological disorder characterized by a mutation in the β-globin gene and inherited as an autosomal recessive disorder. SCD was initially endemic in areas affected by malaria, but migrations have raised its prevalence in other areas where it was previously uncommon (Roberts and de Montalembert, 2007). In Europe, 1-5 per 10,000 people are estimated to suffer from the disease (Orphanet, 2025). Due to the underlying vascular damage, most patients experience lifelong morbidities, resulting in acute complications (e.g., acute chest syndrome and vaso-occlusive crises [VOCs]) and chronic injury to multiple organs including the brain, kidney, and the cardiopulmonary system (Ware et al., 2017). VOCs are acutely painful events that constitute the primary cause of emergency room visits and inpatient admissions and are also associated with the occurrence of serious complications and early mortality (Shah et al., 2019; Lobo et al., 2018; Baile et al., 2019).

SCD significantly shortens the patient’s life expectancy and causes a considerable number of deaths in children under 5 years old in low- and middle-income countries (Lubeck et al., 2019; Wastnedge et al., 2018). The disease has a negative impact on quality of life (QoL) in both children and adults (Osunkwo et al., 2021; Panepinto and Bonner, 2012). Quality-adjusted life expectancy is reduced by half, with a difference of 34 years of quality-adjusted life expectancy between individuals with and without SCD (Lubeck et al., 2019). Absenteeism and productivity losses caused by disability and hospital admissions also reduce the patient’s financial resources and pose an important economic burden not only on the family nucleus but also on society (Holdford et al., 2021; Huo et al., 2018).

A significant barrier faced by SCD patients is the issue of healthcare equity, particularly in the context of racial and ethnic diversity. Studies have shown that patients from minority backgrounds often experience disparities in SCD care (Lee et al., 2019), which can be attributed to factors such as unconscious racial biases within healthcare systems (Anderson et al., 2023). These biases can lead to differences in treatment approaches, pain management, and overall quality of care received by patients (Anderson et al., 2023; Power-Hays et al., 2020). The integration of diversity and inclusion practices in SCD care is crucial in addressing these disparities and ensuring equitable healthcare for all patients, regardless of their background (Hematology, 2023).

Interventional strategies, including early diagnosis and treatment with regular follow-ups, are necessary to prevent serious complications and to decrease disease burden (Lobitz et al., 2018; Kato et al., 2018). However, despite their severe pain (Coleman et al., 2016), patients with SCD are often undertreated, which has been, at least partially, attributed to physicians’ unconscious racial biases (Power-Hays et al., 2020). Patients have also reported other barriers to care, such as limited physician knowledge or experience, and the physicians’ lack of appreciation of the patient’s SCD knowledge (Phillips et al., 2022). Perceived social support from healthcare professionals (HCP), as well as from friends and family is essential and has a positive effect on patients’ self-care (Matthie et al., 2015).

The use of social media channels has become ubiquitous in the lives of teenagers and adults to generate and share content regarding many contexts, including health (Chen and Wang, 2021). More than 5.2 billion people globally use social media, and this number is expected to rise in the following years (Datereportal, 2025). Stakeholders of chronic diseases (patients, caregivers, health organizations, and health professionals) use social media platforms for different health purposes (Chen and Wang, 2021; Patel et al., 2015). For instance, patients use these platforms to seek and share health-related information and to exchange social support, among others (Chen and Wang, 2021). Twitter (now known as X) and Facebook are among the most popular social media channels for stakeholders of chronic disease in general (Chen and Wang, 2021; Patel et al., 2015).

Specific knowledge on the most popular social media channels and the topics discussed by main SCD stakeholders remains scarce. The analysis of this publicly shared information is a potential research data source that adds a new perspective on aspects assessed by other methodologies, such as surveys or non-interventional studies. Moreover, the insights from a social media listening (SML) approach are particularly relevant in SCD, as patients frequently feel stigmatized and might find difficult to share their experiences in person (Jenerette and Brewer, 2010). The present study aimed to analyze discussion by SCD stakeholders (patients, caregivers, family, friends and HCP) on social media to gain understanding on several topics, including their awareness of the disease, their perceptions of the patient journey, hospitalizations and complications due to SCD, the impact of the disease on QoL, and unmet needs by using SLM across Europe.

## Materials and methods

### Study design and data collection

Data regarding SCD-specific terms were collected retrospectively for 24 months from April 2019 to April 2021 across 14 European countries (the United Kingdom [United Kingdom], Spain, France, Switzerland, Belgium, Germany, Austria, the Netherlands, Italy, Portugal, Denmark, Finland, Norway and Sweden) in the following languages: English, Spanish, French, German, Dutch, Italian, Portuguese, Danish, Finnish, Norwegian, and Swedish. Data were collected from open access blogs, forums, and social networking sites (including Twitter, public Facebook, Instagram, and YouTube). Advanced social media analytics tools were used to conduct searches across countries and to collect and aggregate publicly available data. Talkwalker (2025) was used in all countries except in United Kingdom, where Social Studio (Salesforce, 2025) was used.

### Operational definitions

The data universe in our study refers to the entire collection of social media posts and conversations related to SCD gathered before any filtering or analytical processing. This includes all relevant and irrelevant discussions identified by our search criteria across various platforms.

Patient-centric conversations were defined as any relevant social media post where a patient’s lived experiences were the center of the conversation. This includes direct accounts from patients themselves, as well as discussions by caregivers, family members, and healthcare professionals that center around the patient’s perspective and experience of living with SCD.

QoL refers to the overall wellbeing of individuals living with SCD, including the emotional, physical, social and financial domains. Unmet needs were defined as the deficiencies in support, healthcare, and resources that SCD patients face.

### Data analysis

A 3-tier technique was used to identify relevant data, with random sampling procedures generating the final dataset for analysis. Conversations containing SCD-specific terms were extracted using search strings and social media aggregator tools. The information was filtered to a contextualized dataset by automated relevancy algorithms (containing keyword-based relevancy algorithms) and manual review against pre-defined criteria (Supplementary Table S2). The initial dataset underwent a relevancy check to exclude categories.

Search strings were built in each language to identify SCD-related posts or conversations. Boolean operators (AND, OR) were used to combine individual keywords within the search strings (Supplementary Table S1).

The output from automated relevancy check was then analyzed manually to check if any other irrelevancy had entered the data. The final cleaned dataset was then contextualized by assessing the content for the possibility of answering at least one research question in scope. Once the contextualized data sample was ready, relevant posts were categorized by channel type, by country, and when possible, by stakeholder, based on the language used in the post.

The final analysis dataset was manually coded by the team of analysts, who went through each of the sampled posts and segregated them based on the references to the mentioned categories. A deep-dive analysis was performed on the filtered data sets to further analyze insights and topics relating to stakeholders’ perceptions of multiple aspects of SCD. To decrease the risks of biases related to manual analysis (e.g., the analyst’s perception of the content being analyzed, which might interfere with what was classified as negative, positive, or neutral, and his/her judgement on sentiments), the analyzed data were validated through multiple quality checks by more than one analyst.

Most outcomes from patient-centric conversations were analyzed using descriptive statistics and have been reported with numbers/percentages; however, some results were insights that were inferred from conversations by the analysts, hence, no percentages or numbers were associated with these outcome measures. Analyses presented here aggregate across all stakeholder groups unless otherwise specified.

Users’ gender was determined through the identification of indicative lexicon mentions such as gender-associated suffix and prefix and reference to a relation (e.g., wife, daughter, husband, father, etc.). User ages were identified by regular expressions of age.

A total of five data analysts conducted data analysis in this study. All of these professionals completed the necessary pharmacovigilance and governance training applicable to SML programs, as per Novartis guidelines.

### Patient confidentiality

All data utilized and presented in the study were obtained from publicly accessible sources without accessing password-protected information. The pharmacovigilance requirements were secured for the conduct of this study. All data was de-identified and anonymized and posts that are reproduced verbatim have not been included.

## Results

### Posts overview

**FIGURE 1:**
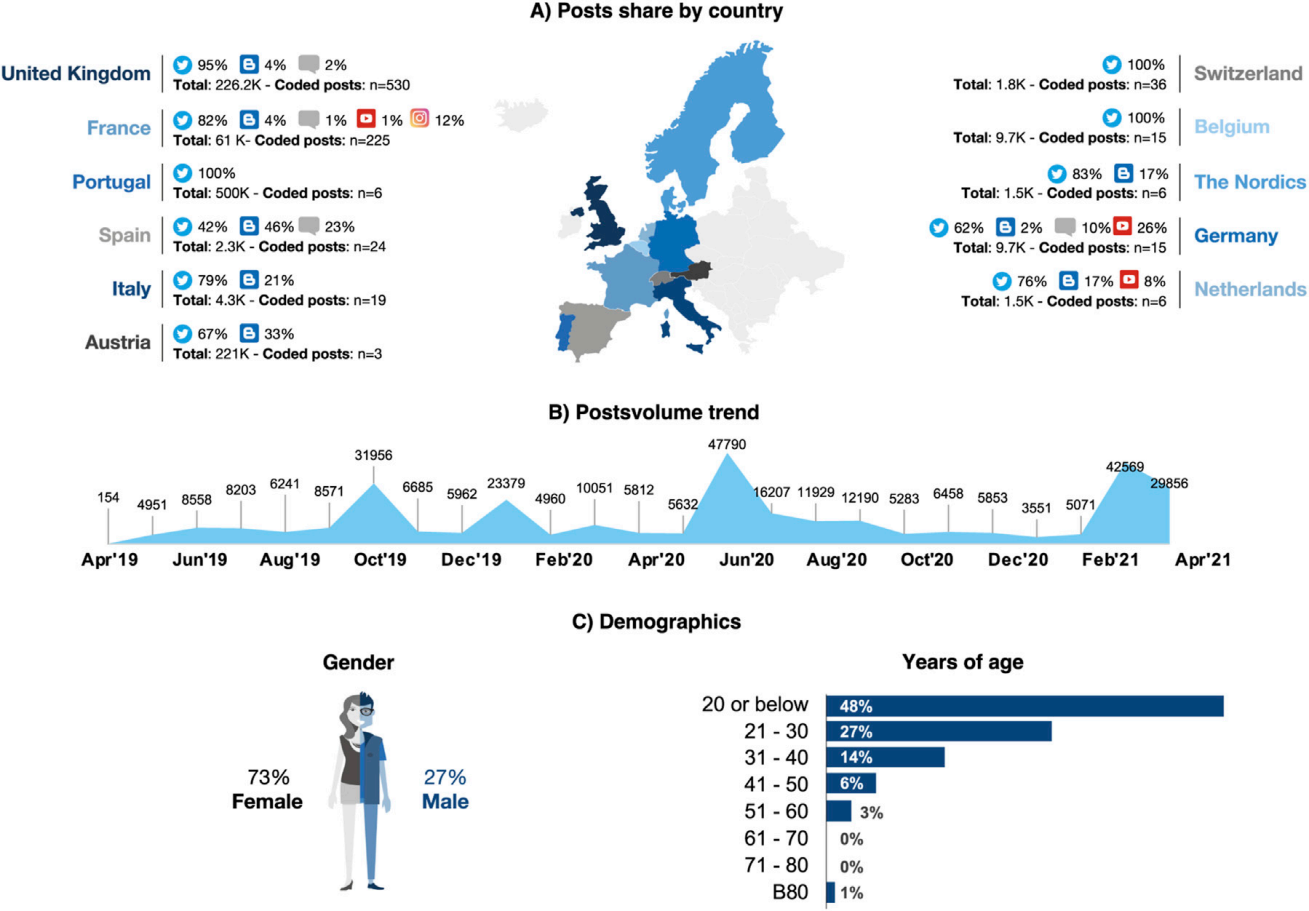
Data source and origin of relevant posts **(A)** Country of origin of posts (data universe, n = 317.900; relevant conversations coded posts, n = 945); **(B)** Posts volume trend (data universe: n = 317.900); **(C)** patient demographics (gender; n = 744; years of age, n = 154).

During the study period, 317,872 posts related to SCD were detected on social media channels. The highest share in posts came from the United Kingdom (71%) followed by France (19%), with the rest of the countries contributing ≤2% of the conversation share (Figure 1A). A peak of posts occurred in June 2020 (Figure 1B). #SickleCell was the most frequently used hashtag (44.3k mentions). Hashtags related to specific patient cases, such as #RichardOkorgheye, were also popular (11.4k). The hashtags #SCD (1.4k), #sicklecelldisease (1.4k) and #Thalassaemia (1.2k) were more likely to be used by HCPs or patients highlighting specific topics related to SCD, such as advances in research, or information sessions targeted at SCD patients. Supplementary Table S3 displays the main hashtags used by stakeholders on the different channels.

Of the whole data universe, 945 posts were identified as patient-centric conversations and were analyzed. The highest share in these conversations came from the United Kingdom (56%), followed by France (24%), and Germany (4%) (Table 1). Most relevant conversations were on Twitter (88%), whereas blogs (5%), Instagram (3%), forums (2%), or other platforms (2%) only contributed small volumes. Indeed, Twitter was the only social media with SCD conversations in Portugal, Switzerland, and Belgium (Figure 1A). Patients were the primary stakeholder in all countries (61%) except in Belgium and Austria, where family and friends (53%) or others (including organizations, communities, and patient support groups) (100%) prevailed, respectively (Table 1).

**TABLE 1:** Stakeholders (relevant posts) by country.

	Patients	Friends and family	Caregivers	HCPs	Miscellaneous	N
	%					
EU	61	16	4	3	16	942
United Kingdom	58	15	4	2	21	530
France	74	14	3	2	8	225
Germany	62	7	12	7	12	42
Spain	25	25	0	21	29	24
Italy	63	16	5	0	16	19
Netherlands	61	22	11	3	3	36
Switzerland	47	28	6	8	11	36
Belgium	40	53	0	0	7	15
The Nordics	67	33	0	0	0	6
Portugal	100	0	0	0	0	6
Austria	0	0	0	0	100	3

Miscellaneous Included “others” who were unidentified, organizations, communities and patient support groups.

### Demographic profile

Patients discussing SCD on social media were mainly females (73%) (n = 744). In Spain, Austria, and Germany, men were more visible than women; Switzerland had an almost even gender ratio; and Portugal featured only female patients. In patients where age was available (n = 154), almost half (48%) were 20 years old or younger (Figure 1C). In a few conversations in the United Kingdom, male patients mentioned how hard it was to share their struggles with SCD due to gender stereotypes, such as men having to be strong.

### Patient journey stages

**FIGURE 2:**
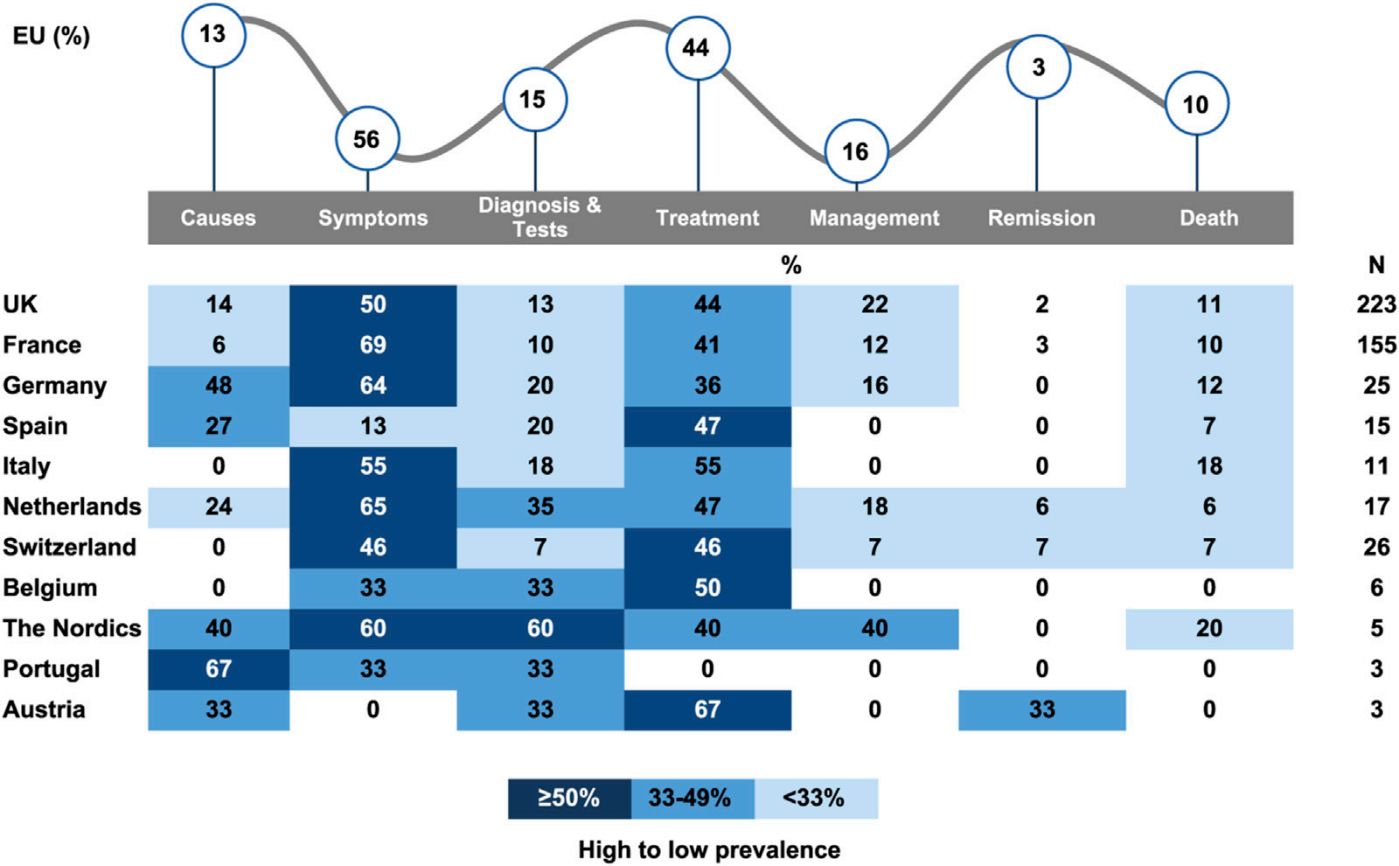
Patient journey stages discussed on conversations (%) Total % may be >100% due to mention of multiple themes in posts.

Patient journey stages were addressed in 52% of all conversations (Figure 2). Symptoms were the most discussed topic (56%), especially pain episodes or pain in general (58%) and VOCs (37%), generally associated with pain (Table 2). In Switzerland, pain was often described as unbearable and excruciating by patients and caregivers; family and friends expressed feeling powerless and crushed by having to stand by and watch a loved one suffer. In the Nordics, pain was described as an almost unbearable, shooting pain in the limbs. It was also mentioned that HCPs failed to acknowledge patient’s pain and effectively treat it, which patients in the United Kingdom attributed to racial bias. Fatigue (8%), mobility issues (4%), and other symptoms (32%) were also discussed.

**TABLE 2:** Conversations about symptoms (patient journey) by country.

	Episodes or pain/pain in general	Acute crisis	Fatigue	Mobility issues	Others	N
	%					
EU	58	37	8	4	32	271
United Kingdom	63	30	8	0	41	110
France	50	47	7	10	22	107
Germany	69	13	0	0	38	16
Spain	100	0	100	0	50	2
Italy	50	33	0	0	17	6
Netherlands	45	45	9	0	27	11
Switzerland	62	54	8	0	38	13
Belgium	50	50	0	0	50	2
The Nordics	67	0	33	33	33	3
Portugal	0	0	100	0	0	1
Austria	NA	NA	NA	NA	NA	0

The second most discussed stage of the patient journey was treatment (44%). Blood transfusions was the most mentioned treatment (34%), followed by pain relieving narcotics (10%), stem cell transplant/bone marrow transplant (9%), other pain relief medications (8%), and supplemental oxygen (4%), among others (Table 3). In the Nordics, blood transfusions was the only treatment mentioned. Generic mentions of treatment (classified by ‘others’ in Table 3) were second most common treatment type and was most visible in Switzerland (77%), France (70%), Italy (67%), Belgium (67%), UK (58%) and the Netherlands (57%). Stem cell transplant was the most visible treatment type the Netherlands (29%) and Belgium (33%), whereas in the United Kingdom, the country with the highest volume of SCD conversation, it appeared in only 2% of the conversations.

**TABLE 3:** Conversations about treatment (patient journey) by country.

	Blood transfusions	Pain relieving narcotics	Stem cell transplant/Bone marrow transplant	Other pain relief medications	Supplemental oxygen	Antimetabolites	Others	N
	%							
EU	34	10	9	8	4	3	62	213
United Kingdom	46	14	2	7	5	4	58	98
France	25	8	13	11	3	0	70	64
Germany	22	22	22	11	0	11	33	9
Spain	57	0	29	0	0	0	43	7
Italy	17	17	0	0	0	0	67	6
Netherlands	14	0	29	0	0	14	57	7
Switzerland	23	0	15	0	15	0	77	13
Belgium	0	0	33	33	0	0	67	3
The Nordics	50	0	0	0	0	0	50	2
Portugal	NA	NA	NA	NA	NA	NA	NA	0
Austria	0	0	0	0	0	0	100	2

Treatment was usually discussed without much detail, and conversations were about efficacy (45%), frequency and dosage (23%), availability and access (15%), treatment duration (15%), side effects (15%), inefficacy (14%), and others (31%) (Supplementary Table S4). Efficacy was often associated with blood transfusions and stem cell transplants, which helped a few patients in the United Kingdom, France, and Switzerland to remit symptoms. In the United Kingdom, campaigns for more blood donations were identified. In Austria and the United Kingdom, patients stated to be cured with gene therapy, after receiving different medications and blood transfusions. One stakeholder mentioned that their blog post about a SCD patient who remitted after receiving stem cell transplant was her/his most popular post. Stem cell transplant was the only treatment positively perceived in more than half of the conversations (53%) (Table 4).

**TABLE 4:** Conversations about treatment (patient journey) by sentiment.

	Positive	Negative	Neutral	N
	%			
Blood transfusions	34	21	45	73
Pain relieving narcotics	13	30	57	23
Stem cell transplant/bone marrow transplant	53	32	16	19
Other pain relief medications	0	31	69	16
Supplemental oxygen	0	0	100	9

Symptoms management was discussed in 16% of the conversations. Avoiding temperature extremes (23%) and coping techniques (22%) were the most mentioned measures, followed by seeking emotional support from communities (19%), more water intake (19%) or avoiding/managing stress (12%). Other aspects of the patient journey discussed were diagnosis and tests (15%), causes (13%), death (10%), and remission (3%). No mentions of recurrence were found. In France and the United Kingdom, conversations around the loss of a patient were often accompanied by appeals either to raise awareness in the hope of discovering an effective treatment, to educate the public about the severity of the illness, or to urge people to be tested for SCD before having children. In the United Kingdom, SCD patients dying due to medical negligence was condemned.

### Hospitalizations and complications due to SCD

Hospital visits/hospitalizations were reported in 11% of the conversations. Of these, 62% had mentions of crises related to symptoms, and 8% were related to the progression of the disease.

Being at high risk for COVID-19 was the most discussed complication (36%), with mentions in almost all countries (Table 5). Pregnancy complications such as patients suffering multiple miscarriages were the second most mentioned complication (15%). Mental health issues and severe anemia were equally prevalent (7%). Patients in Spain and the United Kingdom talked about the impact of SCD on mental health, often related to episodes of pain or being housebound. Patients also discussed their weaker immune system (6%), or other complications (31%), including bone degeneration (5%), organ damage (4%), or stroke (4%).

**TABLE 5:** Complications due to SCD discussed on conversations.

	High risk for COVID-19	Pregnancy complications	Severe anemia	Mental health problems	Weaker immune system	Others^a^	
	%						N
EU	36	15	7	7	6	51	138
United Kingdom	32	24	4	10	7	34	71
France	29	9	6	0	3	71	35
Germany	33	0	17	0	0	50	6
Spain	47	0	7	13	13	27	15
Italy	100	0	0	0	0	0	1
Netherlands	44	11	33	0	0	11	9
Switzerland	29	0	0	0	0	100	7
Belgium	100	0	0	0	0	0	1
The Nordics	0	0	0	0	0	100	1
Portugal	0	0	0	0	0	100	1
Austria	NA	NA	NA	NA	NA	NA	0

^a^ Including eye problems, organ damage, sepsis, stroke, gallstones, gum and teeth problems, avascular necrosis, vaso-occlusive crises (VOCs), and other.

### Quality of life

**FIGURE 3:**
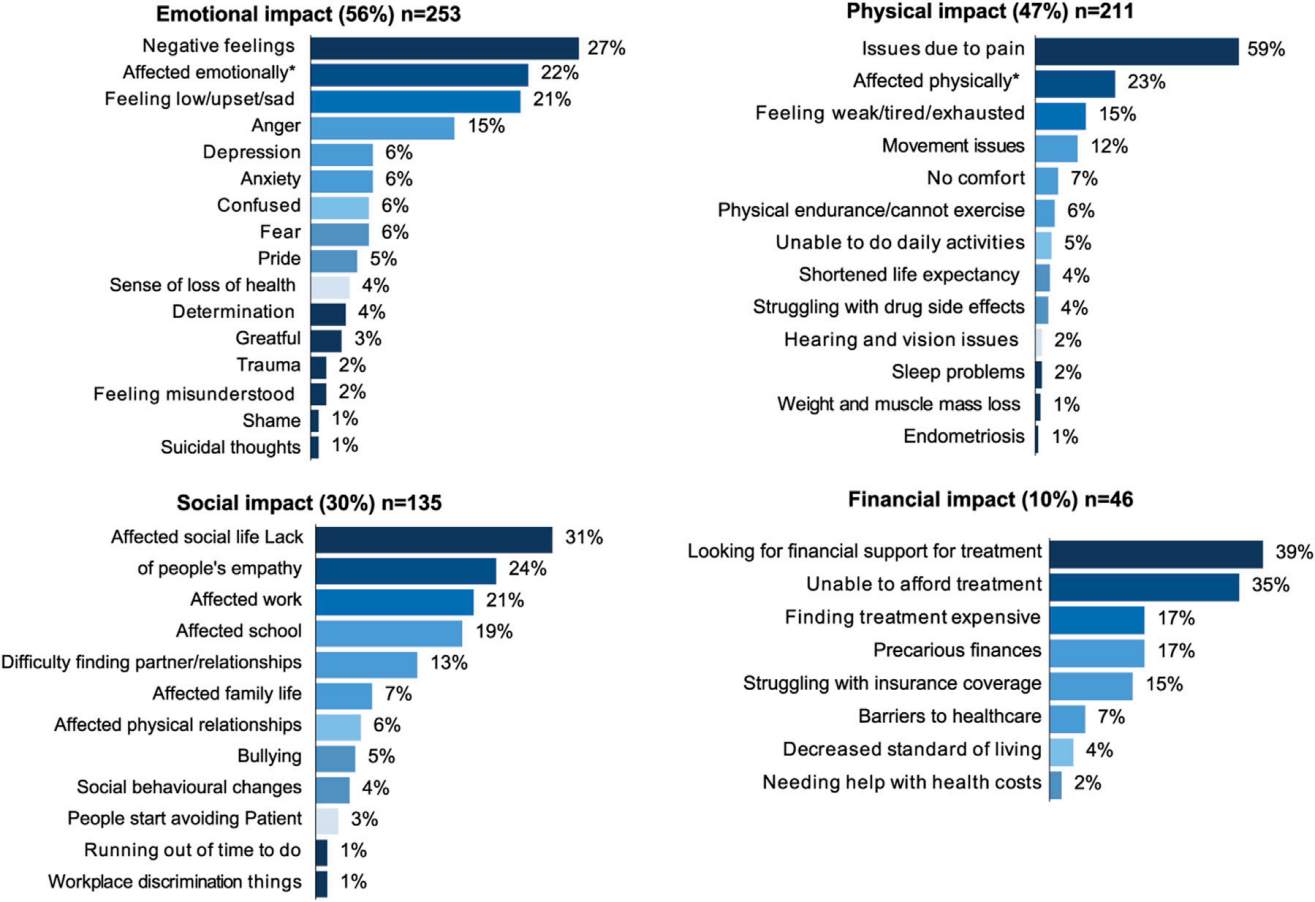
Topics discussed regarding the impact of SCD on quality of life.

Impact of SCD on QoL was also extensively discussed among patients (45%). Figure 3 presents specific topics discussed within all the QoL dimensions. Emotional impact was discussed in 56% of the conversations. Negative feelings and being affected emotionally in general were mentioned to be triggered by repeated hospital visits, lack of awareness and understanding from HCPs and the general public, and having to deal with symptoms, such as debilitating pain. Feeling low/sad was also prevalent and often related to pain or the lack of empathy from others. Depression was associated with feeling limited by SCD or being ostracized and having a shortened life expectancy. Positive feelings such as pride, determination, and gratitude were also occasionally visible, as patients acknowledged their strength and were grateful for support.

Physical impact was the second most discussed QoL dimension (47%). Physical pain dominated conversations and was often described as unbearable and debilitating; tiredness/fatigue/weakness and movement issues were prevalent across all countries; pain was the main issue affecting patients’ lives and wellbeing. The pain was described as “pure hell” or “too strong to put into words” by patients.

Social impact of SCD was found in 30% of the conversations. Patients frequently saw their social life affected (31%) by the unpredictable nature of SCD crises. For example, a patient in Italy complained about having to miss social activities because of the strong pain, and other patient in Sweden attributed missing social events due to long hospitalizations. Occasionally, social life was also affected by shielding due to COVID-19, such as in the United Kingdom and Spain. Lack of empathy and understanding from others was frequently encountered. Patients described being perceived as “impostors”, “drug addicts”, or “lazy”, and having their suffering questioned or dismissed, even by HCPs. Occasionally, this even led to medical negligence and malpractice, such as in Switzerland, where a patient was denied medication from a HCP who did not believe he was in pain. In the United Kingdom and Germany, patients mentioned that when they missed work or school due to SCD crises, they were questioned.

**FIGURE 4:**
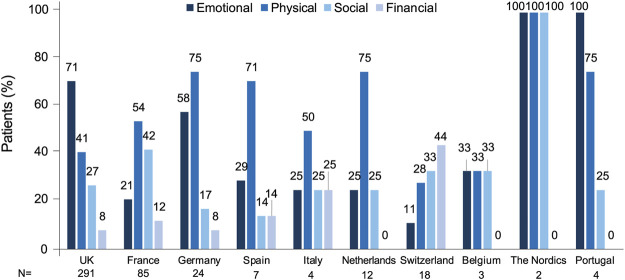
Quality of life impact by country.

Financial impact was the least discussed QoL domain (10%). Overall volumes on the financial impact were low. Stakeholders frequently use social media to raise funds for themselves or on behalf of other patients who were unable to afford treatment. Particularly in Switzerland, several patients sought financial support to cover treatment by either asking for donations or selling handmade items. Patients in the United Kingdom expressed their anger at having to cover the costs of life-saving medications themselves. They shared petitions to include SCD in the list of illnesses eligible for a MedEx and said that this was a form of medical racial injustice, since most SCD patients have African ancestors. In Italy, an organization asked for financial support on behalf of patients who could not afford expensive blood transfusions. QoL impact by country is shown in Figure 4.

### Unmet needs

**FIGURE 5:**
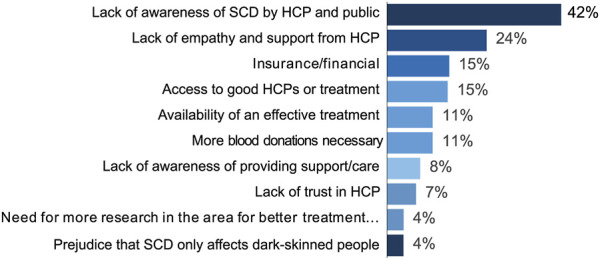
Unmet needs.

Unmet needs were detected in 24% of the conversations. As Figure 5 shows, most frequent unmet needs were lack of awareness of SCD by HCPs and the general public (42%), and lack of empathy and support from HCPs (24%). Patients commented that HCPs did not have enough knowledge and competence in SCD treatment, which was often attributed to racial bias. Patients in the United Kingdom, Switzerland, and the Nordics reported being dismissed and denied treatment by HCPs, which they attributed to the prejudice that patients seeking analgesics are drug addict. In France, lack of awareness was also mentioned related to the lack of genetic tests before conception to prevent passing on the disease to their children. Patients were also mentioned the general public lack of awareness, which forces them to fulfil expectations of normalcy or being bullied.

Discussions on lack of awareness also included mentions of lack of empathy and support from HCPs. Patients in the United Kingdom, France, the Netherlands, and Germany reported having their symptoms dismissed or being mistreated in hospitals, which they attributed to racial bias. A patient in the United Kingdom mentioned associating anxiety with hospital visits, as they were never sure whether HCPs would believe them.

Financial issues were mentioned as an unmet need in 15% of the conversations. In Switzerland, some patients were unable to cover the costs of treatment and fundraising campaigns were conducted by family members, friends, or organizations. In France and the United Kingdom, stakeholders condemned that patients had to pay for life-saving medications themselves. Likewise, a few stakeholders in the United Kingdom noted that SCD needed to be recognized as a disability, as this would allow patients have access to financial support.

The absence of an effective treatment and the need to have access to trained HCPs was also mentioned in 15% of the conversations. In Germany, caregivers and patients expressed frustration over the lack of an effective treatment and the need for more research.

## Discussion

The present study shows that SML can provide novel insights into the experiences and perceptions of SCD stakeholders, including patients, family and friends, caregivers, and HCPs. Symptoms, treatment, and emotional impact of the disease were extensively discussed in the conversations on social media.

To the best of our knowledge, this is the largest SML study ever conducted on SCD, in terms of both the number of analyzed posts and the number of included countries. As far as we are aware, only two previous studies on SCD using SML, one in the US that analyzed 50 posts and one in the United Kingdom that analyzed 513 posts, have been published to date (L et al., 2016; Shastri et al., 2023). Our findings align with and extend existing literature on SCD, particularly regarding the challenges faced by patients in managing their condition and interacting with healthcare systems (Coleman et al., 2016; Adam et al., 2017; Bulgin et al., 2018; Haywood et al., 2009).

SML findings complement information provided by other real-world data sources and make it possible to detect issues that are more difficult to record on studies using traditional research approaches. For instance, while data on patient’s feelings or important experiences can be spontaneously shared in real time on the chosen platform, traditional research methodologies require the presence of the HCP or the use of pre-specified platforms, hampering the collection of data on relevant aspects to patients. Indeed, the use of social media as a source for collecting information on symptoms and disease impact from the patient’s perspective was proposed by the Food and Drug Administration (FDA) in 2018 (Unit ed States Food and Drug Administration FDA, 2022a). Moreover, the FDA is exploring the value of social media mining for earlier detection of rare and serious adverse events (United Stared Food and Drug Administration FDA, 2022b).

Since SML studies analyze information from public sources on social media platforms, they avoid several logistical challenges of traditional research, allowing for accelerated and cost-effective data collection (McDonald et al., 2019). However, they pose unique ethical challenges, since participants do not formally consent to their data being used in the study (Samuel and Buchanan, 2020). Despite the absence of current guidelines on consent or anonymity for social media research, it is recommended that data collected be used to answer specific research questions and presented in an anonymous manner (Moreno et al., 2013). As SML studies become more common (Samuel and Buchanan, 2020; Ro et al., 2022; Faust et al., 2022; Picone SI et al., 2020), more guidelines to conduct this type of studies are expected to be available.

Twitter was, by far, the most popular social media platform for information sharing between SCD stakeholders. In recent years, Twitter has become one of the most important social media platforms in healthcare communication, with an increasing number of patients and HCP professionals sharing a wide range of experiences there (Bennett et al., 2022; Pershad et al., 2018). Most SCD patients were young and female. The age range of the patients did not come as a surprise since young people are the main users of social media platforms (Eurostat, 2025). However, the gender share observed here does not entirely correspond with the overall gender distribution in social media platforms, where users are predominately male (Sproutsocial, 2022), or with the prevalence of the disease itself, where the gender distribution is equal (Reeves et al., 2019). The gender disparity in conversations on SCD might be, at least in part, due to gender stereotypes, such as men having to be strong and not showing vulnerability. In fact, male patients mentioned in some conversations how hard it was to share their struggles with SCD due to these stereotypes. This finding is in line with previous studies showing that women express more personal issues in social networks than men (Voillot et al., 2022).

The study highlighted the desire of patients and caregivers to discuss SCD-related topics and increase public awareness and access to information. Peak of posts was in June 2020, coinciding with the World Sickle Cell Awareness Day (June 19th). At least half of the conversations were around the patient journey, with symptoms being the most discussed topic. Within the context of symptoms, conversation manly focused on pain (acute episodes and pain in general) and acute crises. Pain was not only the most discussed symptom but also the most mentioned factor having an impact on the physical domain of their QoL. In line with this, the Sickle Cell World Assessment Survey (SWAY) of 2145 SCD patients showed that, in high income countries, SCD patients had pain 2.8 days per week on average and a median of 4 VOCs during the previous year (Osunkwo et al., 2021). Almost a quarter of these VOCs were managed at home due to a previous poor experience at the hospital (Osunkwo et al., 2021), highlighting the need to improve management of VOCs.

The most frequently mentioned unmet needs cited in conversations were lack of SCD awareness by HCPs and society and lack of empathy from HCPs, which has been reported in previous studies (Bulgin et al., 2018; Labbé et al., 2005; Renedo et al., 2019; Blakey et al., 2023). In minority ethnic groups, poorer pain management, less respectful behavior, and undertreatment have been identified (Haywood et al., 2009; Green et al., 2003), probably reflecting the underlying structural racism that persists in most western societies (Power-Hays et al., 2020). The stigma of SCD patients has a negative impact on the psychological, physical, and social wellbeing and impairs healthcare interactions and clinical outcomes (Bulgin et al., 2018; Hall et al., 2015). In April 2019, a 21-year-old black patient in the early stages of a sickle cell crisis died at a hospital in the United Kingdom, after he was denied oxygen by a HCP. We found mentions by stakeholders in social media platforms criticizing this incident. Patients also mentioned to be perceived as drug addicts and having their suffering questioned. These findings not only highlight the presence of individual-level and cultural prejudice but also systemic racism as shown by the misalignment of SCD patients needs with the priorities of their healthcare teams (Lee et al., 2019; Smith et al., 2022). Acknowledging these interconnected levels—from individual bias to systemic neglect—is the first step to address structural reforms, such as including training programs among HCPs. In this regard, actions to reduce the impact of racism on patients with SCD have been recently proposed in the US and United Kingdom (Power-Hays et al., 2020; Sickle Cell Society and All-Party Parliamentary Group for Sickle Cell and Thalassemia SCTAPPG, 2022). The frequently mentioned unmet need regarding lack of SCD awareness and lack of empathy from HCPs can be also interpreted within the theoretical framework of symbolic capital (Schneider and -Kamp, 2021). Patients in our study frequently described lacking symbolic capital within healthcare settings. The dismissal of a patient’s report of pain, for instance, can be conceptualized as a struggle over symbolic legitimacy, where the physician’s institutional authority (high symbolic capital) overrides the patient’s experiential knowledge.

Fatigue was also a frequently mentioned symptom in social media conversations, confirming the impact of this symptoms on patients QoL previously suggested (Osunkwo et al., 2021). Studies have further showed that fatigue is highly correlated with depression, anxiety and stress (Ahmadi et al., 2018), and interferes with daily activities (Ameri et al., 2014), which may justify its evaluation as an efficacy endpoint in clinical trials (Osunkwo et al., 2021).

Treatment was the second most discussed topic of the patient journey in the conversations. Blood transfusions was most frequently mentioned treatment, which was often associated with efficacy, a feature also attributed to stem cell transplants. Stem cell transplant was the only treatment positively perceived in more than half of the conversations, which was not surprising due to reported remission or absence of symptoms with this treatment (Kato et al., 2018; Bhalla et al., 2023). Stem cell transplantation protocols are improving rapidly, due to advances in gene editing techniques that help in the genome modification of hematopoietic stem cells (Abraham and Tisdale, 2021). One of these techniques is CRISPR, which enables the precise replacement of a specific region of DNA. Some CRISPR-based treatments for SCD have been already approved by the regulatory agencies and others are still under research (Papizan et al., 2020). In conversations in Austria and the United Kingdom, patients shared that they had been cured with gene therapy, mentioning CRISPR. Gene therapy requires specialized centers for patient care, and therefore accessibility of gene therapy across the globe, especially in low-income areas with a high prevalence of the disease, remains a challenge that needs to be addressed (Frangoul et al., 2020; Carvalho et al., 2021).

Discussion on QoL took up almost half of the conversations, with emotional impact as the most affected dimension. Main reasons for the impairment of psychological wellbeing were repeated hospital visits, lack of awareness and support from HCPs and the public and having to deal with symptoms like pain. Results from the SWAY study showed that 60% of patients stated that SCD caused a high negative impact on emotions; depression and anxiety were reported by 39% and 38% of patients, respectively (Osunkwo et al., 2021). The SWAY study also showed that only one-third of patients were receiving professional emotional support, while 62% had a desire to receive any or more of this type of support (Osunkwo et al., 2021). In our study, among conversations on the emotional impact of the disease, mentions to negative feelings were frequently detected, but also, albeit less frequently, depression, anxiety and suicidal thoughts were discussed. The prevalence of depression in SCD has been found to be up to 5 times higher than in the general population, with higher levels of depression associated with lower QoL (Adam et al., 2017). Studies using validated patient-reported outcomes (PROs) have showed that QoL was impaired in patients with SCD (Osunkwo et al., 2021; McClish et al., 2005; Vilela et al., 2012; Rizio et al., 2020). Importantly, patients who claimed to have suffered prejudice in the past had significantly worse QoL than those who did not (Rodrigues et al., 2021). Other factors associated with worse QoL in SCD were older age, female gender, rural residence, low family income, disease-related complications, hospital admissions, and severity and frequency of VOCs (Rizio et al., 2020; Amr et al., 2011; Dampier et al., 2011).

Financial issues were also mentioned as affecting their QoL and being an unmet need, although to a lesser extent than emotional, physical, and social aspects. Most of the countries where conversations took place have healthcare systems with a wide coverage of the disease, resulting in a lower cost for patients compared to other regions in the world (Johnson et al., 2022). However, some mentions of patients having to pay for life-saving medications themselves were found in France and the United Kingdom. Furthermore, SCD has indirect costs to patients, owing to high unemployment rates and difficulties keeping a job due to absenteeism and productivity loss (Osunkwo et al., 2021; Idowu et al., 2014), frequently caused by VOCs (Rizio et al., 2020). A considerable number of patients mentioned that SCD affected their work or school and a few stated that they even suffer discrimination at the workplace.

Regarding complications due to SCD, a higher risk of COVID-19 was a key concern in conversations. A 4-fold increased risk for COVID-19–related hospitalization and a 2.6-fold increased risk for COVID-19–related death were observed in a large cohort of SCD patients in the United Kingdom using QResearch (a database covering approximately 18% of the United Kingdom population) (Clift et al., 2021). Another study conducted using the same database also showed that SCD was a risk factor for severe COVID-19 outcomes even after one or two doses of COVID-19 vaccination (Hippisley-Cox et al., 2021). It is also important to note that the COVID-19 pandemic itself likely increased social media discussions about SCD since the overall social media use rose during the lockdown (Delogu et al., 2025). During this period people faced higher health risks and had limited healthcare access (Filip et al., 2022). Social isolation during the pandemic may have driven SCD patients to use social media more for support, sharing experiences, and discussing their concerns.

The topics discussed on social media varied across countries. For instance, the emotional impact of SCD was discussed most frequently in the United Kingdom, whereas conversations in France focused more on the physical impact. With our data, we cannot determine whether these differences reflect varying concerns among patients from different countries or cultural differences in the type of information shared on social media platforms.

In contrast, the consistency of topics among countries could be due to the presence of echo chambers (Brugnoli et al., 2019; Scheibenzuber et al., 2023; Cinelli et al., 2021; Papacharissi, 2012), where patients, caregivers and other stakeholders with similar experiences congregate online, reinforcing shared perspectives on SCD symptoms and challenges, stigmatization, and unmet needs. Additionally, performative behavior on social media platforms, where users may modify their expression to align with perceived community expectations or to maximize engagement, could have also influenced the authenticity of shared experiences (Kaplan, 2024).

Overall, our findings provide a comprehensive overview of the issues of interest and concern to SCD stakeholders, mainly patients. SML presents as a relevant tool to explore on first-hand the real-time feelings, perceptions, and experiences of people affected with SCD when they spontaneously choose to share these issues. Studies using this methodology are expected to increase, since they provide complementary information to that obtained using traditional research methods on the patient journey, patient QoL and unmet needs, among other aspects.

The findings from the present study could be useful for improving SCD care at multiple levels. Healthcare providers and policymakers could address the lack of awareness and racial bias in treatment by implementing anti-stigma and cultural competence training, improving pain management protocols, and integrating mental health support into SCD care. Furthermore, adopting patient-centric models—such as multidisciplinary SCD Units that combine in-person and telehealth consultations with psychosocial support—and launching public awareness campaigns, co-designed with patient advocacy groups, would help meet the emotional and social needs identified in SCD conversations. Establishing SML as a continuous monitoring tool could further support timely responses to the concerns of the stakeholders, the development of patient-reported experience measures (PREMs), and guide healthcare policies.

### Limitations

The present study has several limitations inherent to social media research. First, self-selection bias might have occurred since participants in the social media platforms could have specific demographic, socioeconomic or clinical characteristics and the willingness to participate in these platforms. This bias could result in an overrepresentation of younger individuals, those with higher socioeconomic status, or those with less severe disease who may find it easier to engage in social media discussions. Even though this might have hindered the generalizability of the results, the current study included a wide range of social media platforms and a high number of European countries, which might have partially mitigated this potential bias. However, over 90% of the conversations were drawn from Twitter, which may have introduced a platform-specific bias. On this platform, interactions are limited by a maximum character count, which decreases the depth of user interactions compared to other platforms. Future studies should aim to broaden the range of social media platforms and include more conversations on other platforms where discussions are longer, deeper and with more context. Also, hybrid approaches that combine SML with offline methods, such as surveys or focus groups on clinical settings, could increase inclusivity.

Second, it could also be argued that the veracity of the information shared on posts was not verified; however, the benefit of sharing false information in this context seems unlikely, since the aim of posting health-related information is usually to share feelings and experiences within the community, increase awareness and find peer support.

Third, the potential for echo chambers and performative behavior on social media platforms may limit the diversity of perspectives and topics captured in our study. However, these phenomena are less likely in the discussions included in our study (i.e., disease-specific discussions on experiential knowledge) than in other discussions with more polarized views (Cascini et al., 2022). The focus on specific languages and cultures may also omit valuable insights from broader stakeholder groups. Changes in social media algorithms and data access policies over time can affect data availability and consistency. However, these phenomena are inherent to social media platforms and did not invalidate the discussions among stakeholders included here. Furthermore, private conversations, which could offer different views, were not captured in our analysis.

Also, our analysis did not apply an intervention-focused theoretical framework such as the behaviour change wheel (Michie et al., 2011). However, the structured coding schema used here allows for future mapping of these findings to such models, which could support the development of targeted health policies and interventions.

Lastly, the unstructured nature of the data also led to variation in the sample size for each study variable; this, together with the variety in number of posts from different countries, did not allow direct comparisons of conversation content between countries. These factors could limit the generalizability of our findings and suggest the need for caution in interpreting the results. To validate the results of our study, future studies could include information from SML with clinical data and PROs obtained in real-world evidence studies.

## Conclusion

This study offers, for the first time, information on the experience of different SCD stakeholders using the SML methodology across Europe. Conversations occurred mainly on Twitter, by young and mostly female participants. Stakeholders across countries emphasized the need for more awareness of SCD by both HCPs and the general public and the lack of support from HCPs. Patients with SCD actively campaigned to raise awareness of the disease. Discussions were mainly focused on patient journey, particularly symptoms and treatment, and on the negative impact of SCD on QoL, especially in the emotional domain followed by the physical domain. This approach provides exploratory insights to understand the situation of people living with SCD that could help to develop disease management strategies, inform health policies, and design future clinical studies.

## Data Availability

The original contributions presented in the study are included in the article/supplementary material, further inquiries can be directed to the corresponding author.
