# Supplementary material for: Understanding stakeholders’ experience with sickle cell disease by social media listening across Europe
Source: Front Genet. 2025 Sep 29;16:1629510. doi: 10.3389/fgene.2025.1629510 (PMC12516078; doi:10.3389/fgene.2025.1629510)
Supplement: Supplementary file 1 [file Supplementaryfile1.docx]

# Supplemental Digital Content (SDC)

## SDC, Table 1 Search strings

| **Languages** | **Search terms used** |
| --- | --- |
| **English** | (sicklecell OR "sickle cell" OR "#sicklecell" OR "HbS disease" OR "Hemoglobin S disease" OR "sickle-cell" OR "#sicklecellanemia" OR sicklecellanemia) |
| **Italian** | (sicklecell OR "sickle cell" OR "#sicklecell" OR "HbS disease" OR "Hemoglobin S disease" OR "celula falciforme" OR "cellule falciformi" OR drepanocítica OR "anemia a cellule falciformi" OR "anemia falciforme" OR falciforme OR #AnemiaFalciforme OR #AF OR #anemiafalciforme) |
| **Spanish** | ((sicklecell OR "sickle cell" OR "#sicklecell" OR "HbS disease" OR "Hemoglobin S disease" OR celulasfalciformes OR "enfermedad de células falciformes" OR "enfermedad drepanocítica" OR "drepanocitosis" OR sekelsel OR "drépanocyt*" OR "hématie* falciforme*" OR "cellule* falciforme*" OR #drépanocytes OR #hématiesfalciformes OR "trouble drépanocytaire" OR "trait drépanocytaire" OR "anemia falciforme" OR "anemia drepanocítica" OR "crisis drepanocítica" OR "anemia de celulas falciforme" OR #sicklecell OR #sicklecell101 OR #sc101es OR #sicklecellanemia OR #sicklecelldisease OR #AF OR #celulasfalciforme OR #anemiafalciforme OR #enfermedaddeanemiafalciforme OR #rasgodeanemiafalciforme) NOT ("desecho social")) |
| **French** | ("drépanocyt*" OR "hématie* falciforme*" OR "cellule* falciforme*" OR #drépanocytes OR #hématiesfalciformes OR "trouble drépanocytaire" OR "trait drépanocytaire" OR "anémie à hématies falciformes" OR "anémie des cellules falciformes" OR (maladi* NEAR/4 "hématies falciformes") OR "anémie falciforme" OR "anémie drépanocytaire" OR "maladie drépanocytaire" OR "drépanocytose" OR "sicklecell" OR "sickle cell" OR #sicklecell) |
| **German** | (Sichelzell* OR Sichelzellkrankheit* OR Sichelzellanämie* OR "Sichel Zelle" OR "#Sichelzelle" OR Sichelzellenanämie OR "sicklecell" OR "sickle cell" OR "#sicklecell" OR "HbS disease" OR "Hemoglobin S disease" OR Drepanozytose) |
| **Dutch** | ("sicklecell" OR "sickle cell" OR #sicklecell OR "sickle-cell" OR sikkelcelziekte OR sikkelcelanemie OR sikkelcel OR "sikkel cel ziekte" OR "sikkel cel" OR "sikkel-cel") |
| **The Nordics (Norwegian, Swedish, Danish and Finnish Combined)** | (sicklecell OR "sickle cell" OR #sicklecell OR "sickle-cell" OR Sicklecellanemi OR sicklecellsjukdomar OR sicklecellsjukdom OR #sicklecellanemi OR #sicklecellsjukdomar OR #sicklecellsjukdom OR sigdcelle OR sirppisol* OR sigdcelle OR sigdcell* OR seglcell* OR sirppisoluanemia) |
| **Portuguese** | ((((doença* OR enfermidade OR anemia OR célula*) NEAR/4 (falciforme* OR drepanocític* OR drepanocitose)) OR drepanocitose OR #anemiafalciforme OR #doençafalciforme OR #doençasfalciformes OR #célulasfalciformes OR #célulafalciforme OR "sicklecell" OR "sickle cell" OR #sicklecell OR "HbS disease" OR "Hemoglobin S disease" OR "sickle-cell") NOT essay) |

## SDC, Table 2 Pre-defined criteria

| What are the key conversations across social media in the SCD disease space?  What are the SCD patient demographics across the countries in scope?  What is the patient behavior across various stages of the patient journey?  What are the treatment patterns discussed by patients and caregivers?  What are the key channels driving social conversations around SCD?  What are the preferred treatment outcomes being discussed in these conversations?  What is the impact of SCD on patients’ quality of life?  What are the identified pain points/unmet needs highlighted by patient conversations? |
| --- |

## SDC, Table 3 Hashtags

| **Hashtags** | **Mentions** | **Stakeholders** |
| --- | --- | --- |
| #SickleCell | 44.3K | 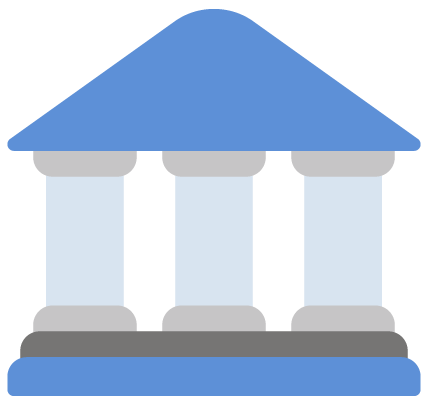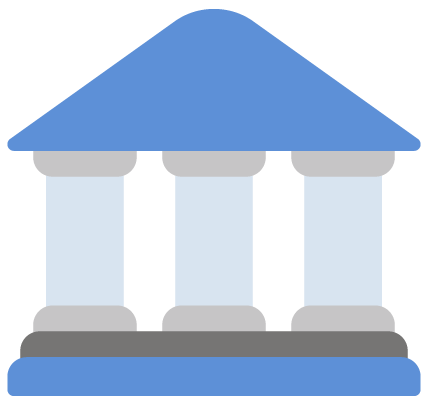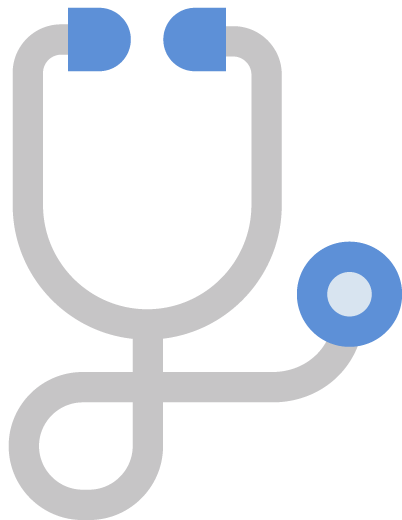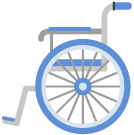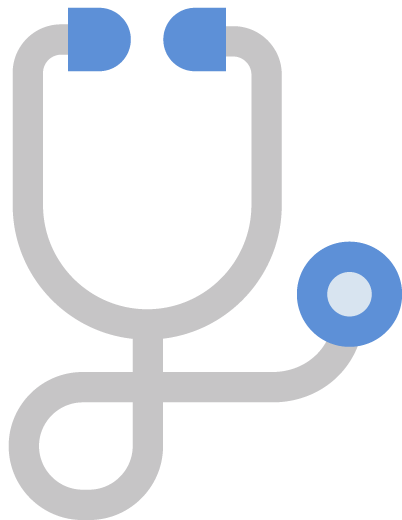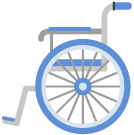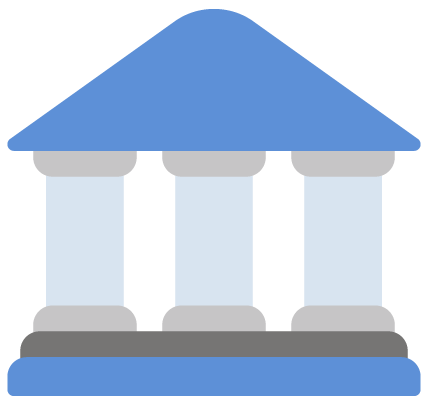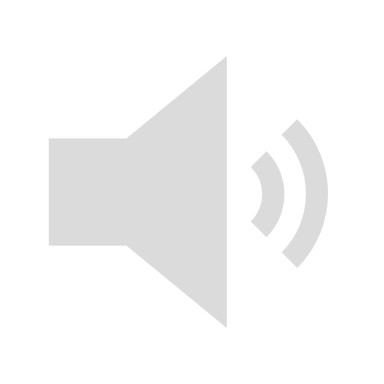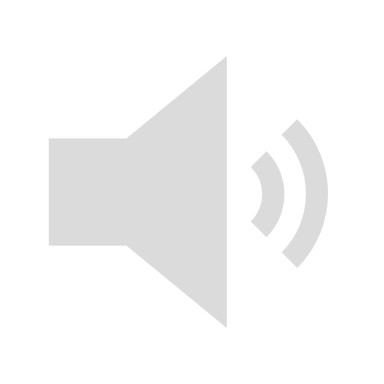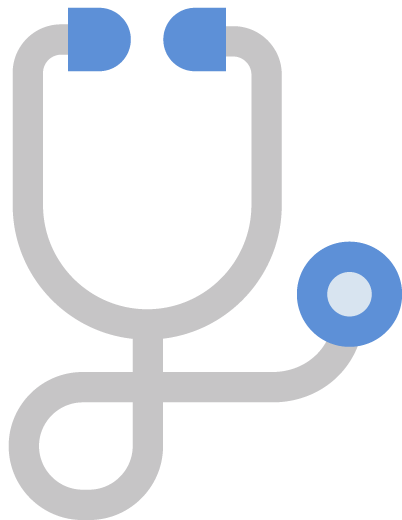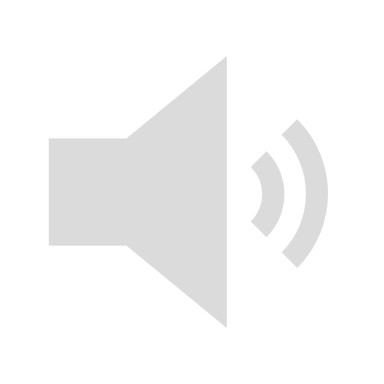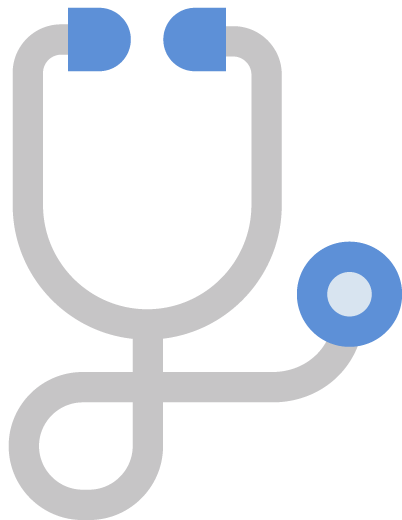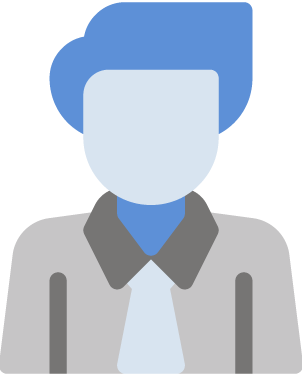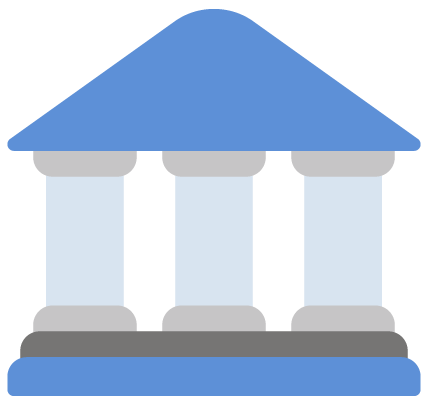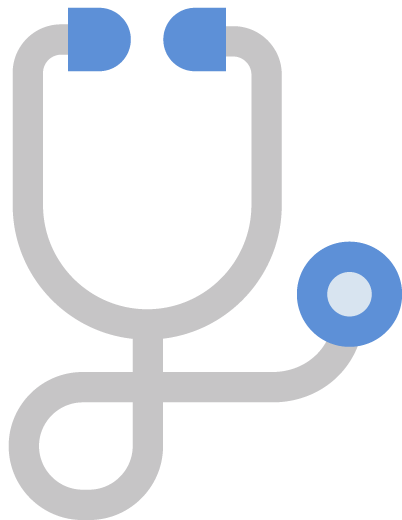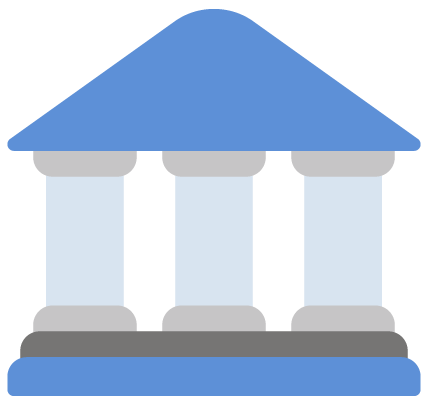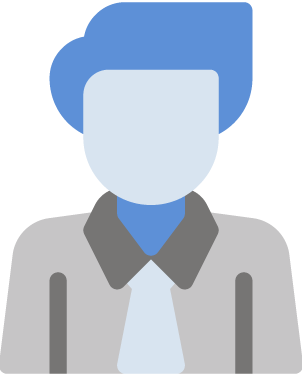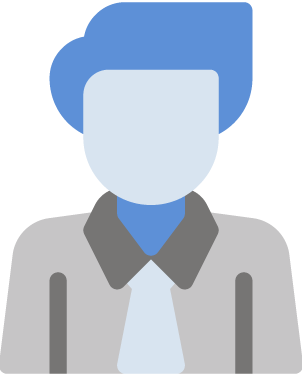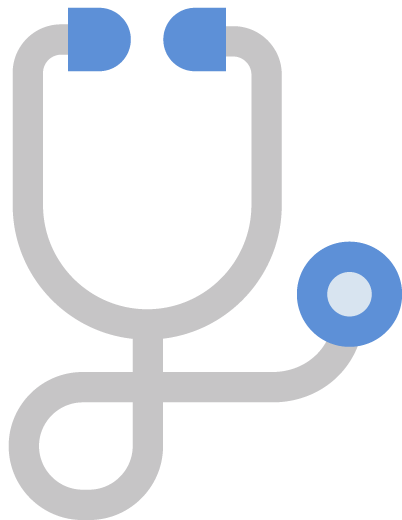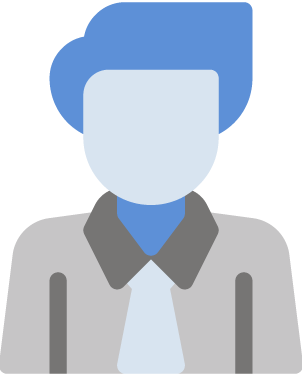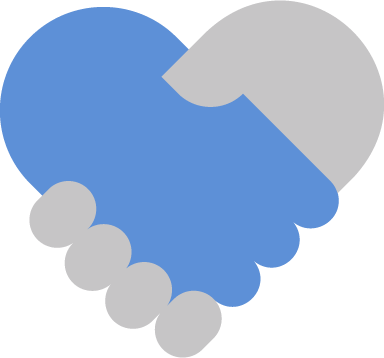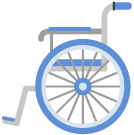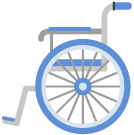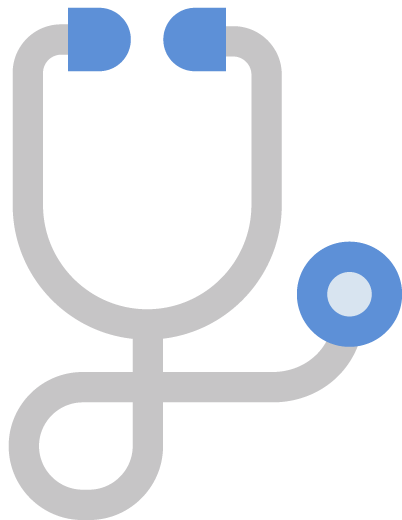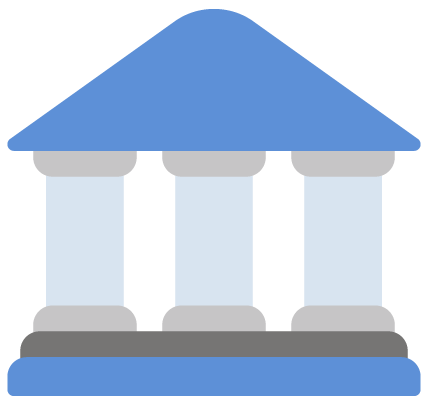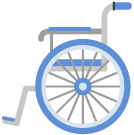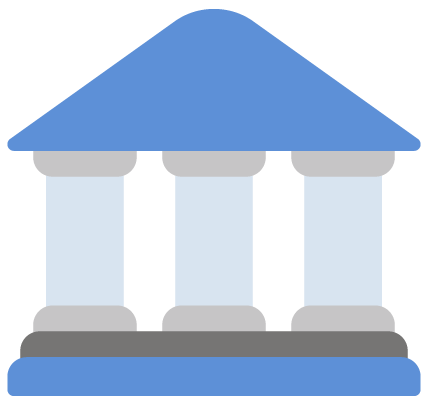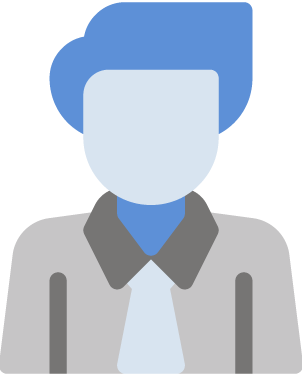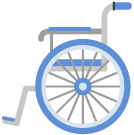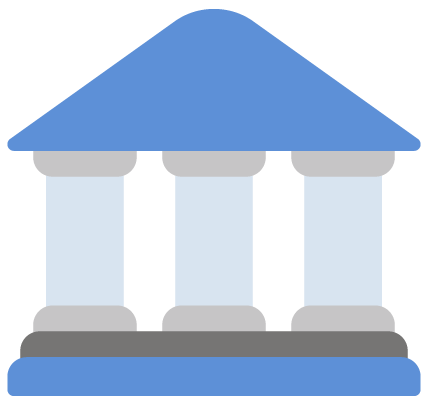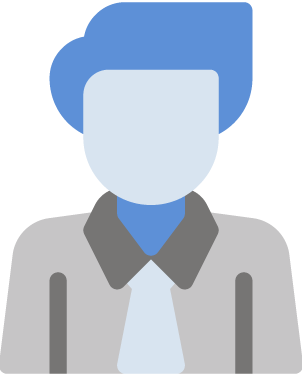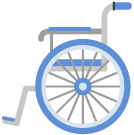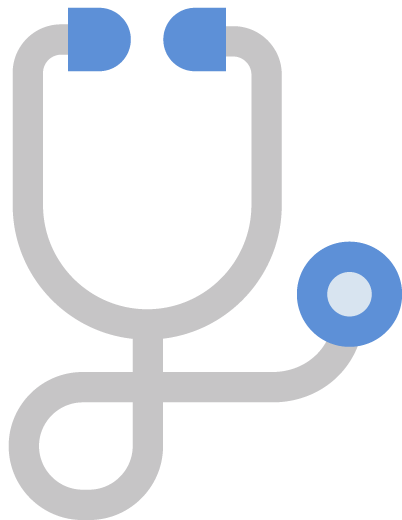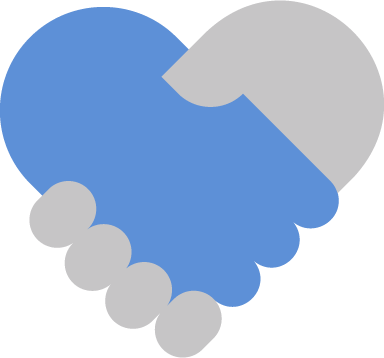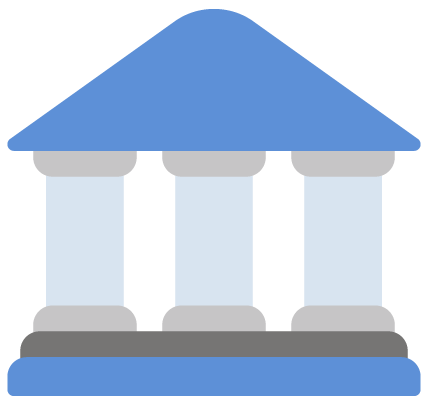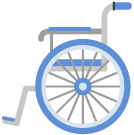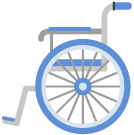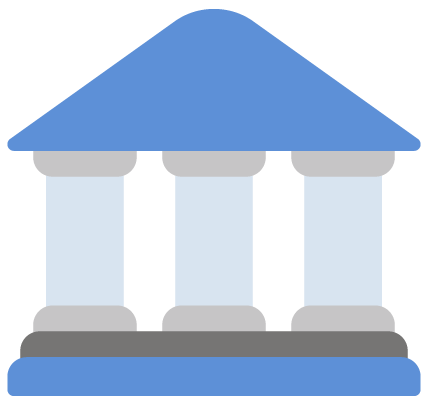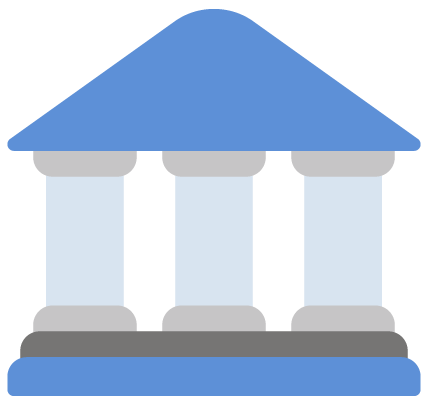 |
| #drépanocytose | 11.4K |  |
| #RichardOkorogheye | 6.5K |  |
| #WorldSickleCellDay | 4.2K |  |
| #SickleCellAwareness | 2.7K |  |
| #GiveBlood | 2.5K |  |
| #SickleCellAwarenessMonth | 1.9K |  |
| #COVID19 | 1.5K |  |
| #SCD | 1.4K |  |
| #sicklecelldisease | 1.4K |  |
| #thalassaemia | 1.2K |  |
| #HerSickledJourney | 1.2K |  |
| #Fundraiser | 1.2K |  |


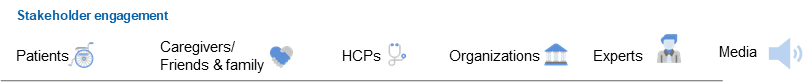


## SDC, Table 4 Treatment features discussed

|  | **Efficacy** | **Treatment frequency and dosage** | **Availability and access** | **Treatment duration** | **Side effects** | **Inefficacy** | **Others** |  |
| --- | --- | --- | --- | --- | --- | --- | --- | --- |
|  | % | | | | | | | N |
| EU | 45 | 23 | 15 | 15 | 15 | 14 | 31 | 108 |
| UK | 49 | 31 | 16 | 12 | 18 | 18 | 35 | 51 |
| France | 32 | 21 | 11 | 25 | 18 | 11 | 32 | 28 |
| Germany | 57 | 29 | 14 | 0 | 0 | 29 | 14 | 7 |
| Spain | 57 | 0 | 29 | 29 | 29 | 0 | 14 | 7 |
| Italy | 33 | 33 | 33 | 0 | 0 | 0 | 0 | 3 |
| Netherlands | 67 | 0 | 33 | 0 | 0 | 0 | 0 | 3 |
| Switzerland | 33 | 0 | 0 | 0 | 0 | 0 | 83 | 6 |
| Belgium | NA | NA | NA | NA | NA | NA | NA | 0 |
| The Nordics | 50 | 0 | 0 | 50 | 0 | 50 | 0% | 2 |
| Portugal | NA | NA | NA | NA | NA | NA | NA | 0 |
| Austria | 100 | 0 | 0 | 0 | 0 | 0 | 0% | 1 |
